# 

**Published:** 1849-10

**Authors:** 


### OUR NEXT NUMBER.

In this we shall give the report on Filing Teeth, submitted by Dr. Whitney, as one of the committee appointed to report on that subject. This committee consisted of Drs. Whitney, Berry, and James Taylor, and after their appointment had no opportunity of consulting together except by letter. Dr. Whitney sent in a report, also Dr. Berry, and it was my duty to compare these, make such additions as I thought necessary, and submit it to the Society. Drs. Whitney and Berry had, however, so well covered the ground, that we considered it best to read their reports, (neither being present,) and discuss the subject at large before the Society. This discussion ended by a request of the Society, that I should, at the opening address, at our next meeting, take up this subject again. Having consented to this, the report sent in by Dr. Whitney will only be published in the next number.

A case of Maxillary Abscess, reported by Dr. Griffith, also an address on Filing Teeth, by Dr. Curtiss, are yet on file. These elicited much discussion.—[J. T.

### TO OUR SUBSCRIBERS.

Those who are in arrears, will have an opportunity, on the receipt of the present number, to remit for two years. We hope this opportunity will be generally complied with, as it is entirely out of the question to send around an agent to make collections, and such a work cannot be sustained without prompt payment.—[J. T. CIN. ED.

